# *N*-[2-(4-Methyl-2-quinol­yl)phen­yl]acetamide: a *P*1 structure with *Z* = 4

**DOI:** 10.1107/S1600536810027650

**Published:** 2010-07-17

**Authors:** F. Nawaz Khan, S. Mohana Roopan, N. Malathi, Venkatesha R. Hathwar, Mehmet Akkurt

**Affiliations:** a Organic and Medicinal Chemistry Research Laboratory, Organic Chemistry Division, School of Advanced Sciences, VIT University, Vellore 632 014, Tamil Nadu, India; b Solid State and Structural Chemistry Unit, Indian Institute of Science, Bangalore 560 012, Karnataka, India; c Department of Physics, Faculty of Arts and Sciences, Erciyes University, 38039 Kayseri, Turkey

## Abstract

The title compound, C_18_H_16_N_2_O, crystallizes in the triclinic space group *P*1, with four independent mol­ecules in the asymmetric unit wherein two mol­ecules have an irregular -*ac*, -*ac*, +*ap* conformation (*ap*, antiperiplanar; *ac*, anticlinal), while the other mol­ecules exhibit a different, +*ac*, +*ac*, +*ap* conformation. The planar (r.m.s. deviation = 0.006 Å in each of the four molecules) quinoline ring systems of the four mol­ecules are oriented at dihedral angles of 32.8 (2), 33.4 (2), 31.7 (2) and 32.3 (2)° with respect to the benzene rings. Intra­molecular N—H⋯N inter­actions occur in all four independent mol­ecules. The crystal packing is stabilized by inter­molecular N—H⋯O and C—H⋯O hydrogen bonds, and are further consolidated by C—H⋯π and π–π stacking inter­actions [centroid–centroid distances = 3.728 (3), 3.722 (3), 3.758 (3) and 3.705 (3) Å].

## Related literature

For the biological activity of quinolines, see: Roopan & Khan (2009 ▶); Roopan *et al.* (2010 ▶); Yates (1984 ▶). For the crystal structures of substituted quinolines, see: Khan *et al.* (2010 ▶); Kushwaha *et al.* (2010 ▶); Subashini *et al.* (2009 ▶). For another crystal structure with *Z*′ = 4 in space group *P*1, see: Bernès *et al.* (2003 ▶).
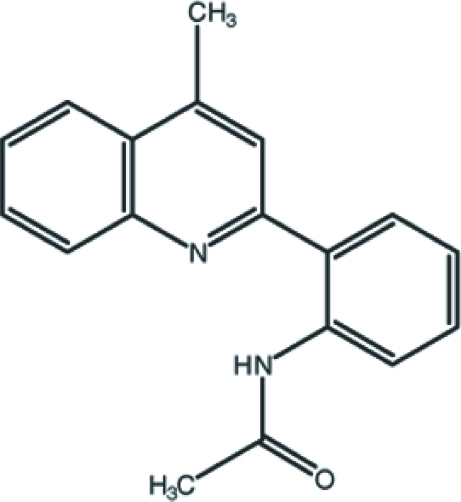


## Experimental

### 

#### Crystal data

C_18_H_16_N_2_O
*M* *_r_* = 276.33
Triclinic, 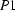

*a* = 9.7383 (5) Å
*b* = 9.9035 (6) Å
*c* = 15.2809 (9) Å
α = 101.414 (5)°
β = 99.188 (5)°
γ = 90.065 (5)°
*V* = 1425.24 (14) Å^3^
*Z* = 4
Mo *K*α radiation
μ = 0.08 mm^−1^
*T* = 295 K
0.20 × 0.10 × 0.10 mm

#### Data collection

Oxford Xcalibur Eos(Nova) CCD detector diffractometer
Absorption correction: multi-scan (*CrysAlis PRO RED*; Oxford Diffraction, 2009 ▶) *T*_min_ = 0.984, *T*_max_ = 0.992
29328 measured reflections
5290 independent reflections
3314 reflections with *I* > 2σ(*I*)
*R*_int_ = 0.079

#### Refinement

*R*[*F* ^2^ > 2σ(*F* ^2^)] = 0.058
*wR*(*F* ^2^) = 0.164
*S* = 0.94
5290 reflections
766 parameters
3 restraints
H-atom parameters constrained
Δρ_max_ = 0.23 e Å^−3^
Δρ_min_ = −0.24 e Å^−3^

### 

Data collection: *CrysAlis PRO CCD* (Oxford Diffraction, 2009 ▶); cell refinement: *CrysAlis PRO CCD*; data reduction: *CrysAlis PRO RED* (Oxford Diffraction, 2009 ▶); program(s) used to solve structure: *SHELXS97* (Sheldrick, 2008 ▶); program(s) used to refine structure: *SHELXL97* (Sheldrick, 2008 ▶); molecular graphics: *ORTEP-3* (Farrugia, 1997 ▶); software used to prepare material for publication: *WinGX* (Farrugia, 1999 ▶).

## Supplementary Material

Crystal structure: contains datablocks global, I. DOI: 10.1107/S1600536810027650/pv2303sup1.cif

Structure factors: contains datablocks I. DOI: 10.1107/S1600536810027650/pv2303Isup2.hkl

Additional supplementary materials:  crystallographic information; 3D view; checkCIF report

## Figures and Tables

**Table 1:** Hydrogen-bond geometry (Å, °) *Cg*3, *Cg*7, *Cg*11, *Cg*15 are the centroids of the C11–C16, C29–C34, C47–C52 and C65–C70 rings, respectively.

*D*—H⋯*A*	*D*—H	H⋯*A*	*D*⋯*A*	*D*—H⋯*A*
N2—H2*N*⋯O4^i^	0.86	2.42	3.181 (6)	148
N2—H2*N*⋯N1	0.86	2.25	2.741 (7)	117
N4—H4*N*⋯O3^ii^	0.86	2.39	3.160 (6)	149
N4—H4*N*⋯N3	0.86	2.25	2.735 (7)	116
N6—H6*N*⋯O2^iii^	0.86	2.51	3.262 (6)	147
N6—H6*N*⋯N5	0.86	2.21	2.703 (7)	116
N8—H8*N*⋯O1	0.86	2.49	3.239 (6)	146
N8—H8*N*⋯N7	0.86	2.20	2.705 (7)	118
C36—H36*B*⋯O3^ii^	0.96	2.31	3.201 (7)	154
C44—H44⋯O2^iii^	0.93	2.60	3.472 (9)	157
C54—H54*B*⋯O2^iii^	0.96	2.28	3.213 (8)	162
C10—H10*A*⋯*Cg*15^iv^	0.96	2.76	3.586 (7)	145
C28—H28*B*⋯*Cg*11	0.96	2.63	3.579 (7)	171
C46—H46*C*⋯*Cg*7^i^	0.96	2.65	3.594 (7)	168
C64—H64*C*⋯*Cg*3^v^	0.96	2.65	3.604 (7)	173

Symmetry codes: (i) 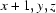; (ii) 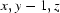; (iii) 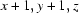; (iv) 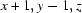; (v) 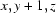.

**Fig. 1.**
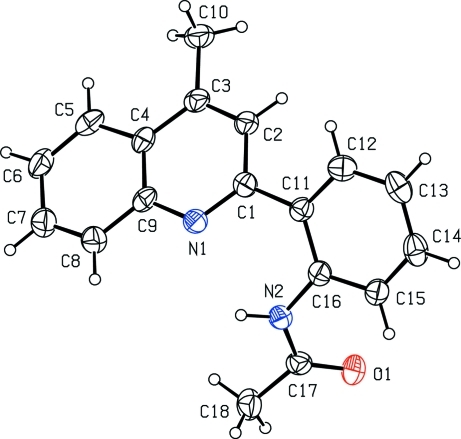
The molecular structure of molecule A of the title compound; displacement ellipsoids drawn at the 50% probability level.

**Fig. 2.**
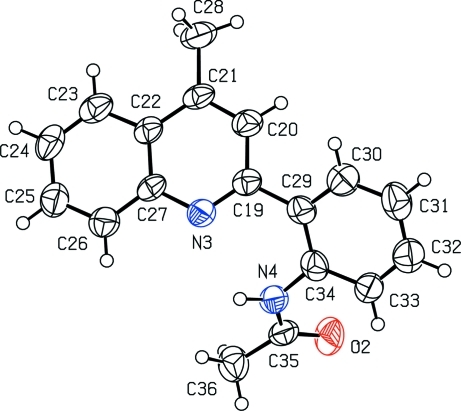
The molecular structure of molecule B of the title compound; displacement ellipsoids drawn at the 50% probability level.

**Fig. 3.**
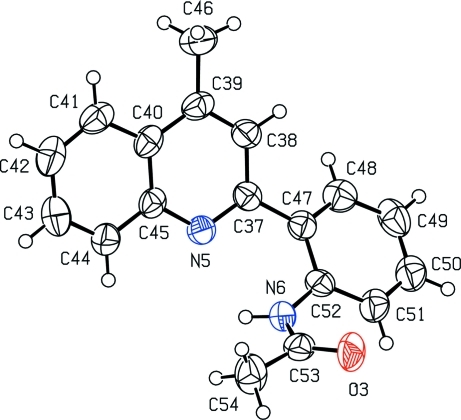
The molecular structure of molecule C of the title compound; displacement ellipsoids drawn at the 50% probability level.

**Fig. 4.**
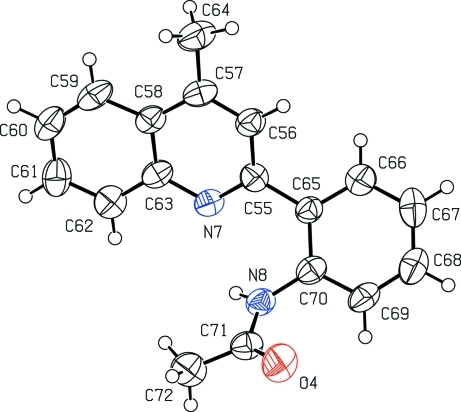
The molecular structure of molecule D of the title compound; displacement ellipsoids drawn at the 50% probability level.

**Fig. 5.**
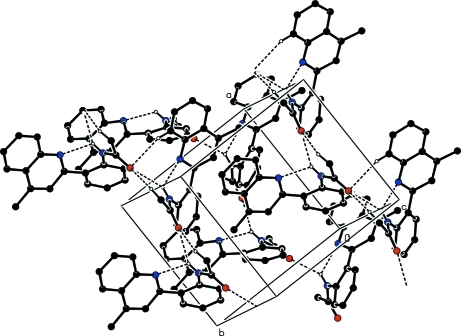
Molecular packing and the hydrogen bonding of the title compound viewed down a axis. H atoms not involved in the intermolecular interactions (dashed lines) have been omitted for clarity.

## supplementary crystallographic information

### Comment

Quinolines and their derivatives occur in numerous natural products, many of which possess interesting physiological and biological properties. Quinoline derivatives have been developed for the treatment of many diseases like malaria, HIV, tumor, and antibacterial infections (Yates, 1984). Since the turn of the century, the development of concise and effective methodologies for the preparation of libraries of small molecules for research in drug discovery has remained a major challenge. A number of strategies have been developed to address this challenge (Roopan *et al.*, 2009, Roopan *et al.*, 2010). We herewith reporting the synthesis of 2-substituted quinoline using 1-(2-aminophenyl)ethanone in acetic acid and drop of sulphuric acid at 453 K.

The title compound crystallizes in the triclinic space group P 1 with four independent molecules, A, B, C and D with O1, O2, O3 and O4, respectively, in the asymmetric unit (Figs. 1-4) with no evidence of pseudo- or non-crystallographic symmetry. Crystals belonging to this class, namely Z' = 4 in space group P 1, are not uncommon (Bernés *et al.*, 2003). The molecules A and B have an irregular -ac, -ac, +ap conformation (C2-C1-C11-C16,  C11-C16-N2-C17, C16-N2-C17-C18 in molecule A and  C20-C19-C29-C34,  C29-C34-N4-C35, C34-N4-C35-C36 in molecule B) while the other molecules C and D exhibit a different, +ac, +ac, +ap conformation (C38-C37-C47-C52, C47-C52-N6-C53, C52-N6-C53-C54 in molecule C and  C56-C55-C65-C70, C65-C70-N8-C71, C70-N8-C71-C72 in molecule D). The planar quinoline ring system of each molecule makes a dihedral angle of 32.8 (2)° for A, 33.4 (2)° for B, 31.7 (2)° for C and 32.3 (2)° for D with the benzene ring [C11–C16 for A, C29–C34 for B, C47–C52 for C and C65–C70 for D] bound to it.

The crystal packing is stabilized by intermolecular N—H···O and C—H···O hydrogen bonds (Table 1, Fig. 5), and are further consolidated by C—H···π (Table 1) and π-π stacking interactions [*Cg*1···*Cg*14(*x*, -1 + *y*, *z*) = 3.728 (3), *Cg*2···*Cg*13 (1 + *x*, -1 + *y*, *z*) = 3.722 (3), *Cg*5···*Cg*10(-1 + *x*, *y*, *z*) = 3.758 (3), *Cg*6···*Cg*9(*x*, *y*, *z*) = 3.705 (3) Å; where *Cg*1, *Cg*2, *Cg*5, *Cg*6, *Cg*9, *Cg*10, *Cg*13 and *Cg*14 are the centroids of N1/C1–C4/C9, C4–C9, N3/C19–C22/C27, C22–C27, N5/C37–C40/C45, C40–C45, N7/C55–C58/C63 and C58–C63 rings, respectively].

### Experimental

About 1 mmol of 2-aminoacetophenone in 10 ml of glacial acetic acid and 2 drops of concentrated sulfuric acid were refluxed at 393 K for 8 h. After the completion of the reaction (monitored by TLC) the mixture was poured in to crushed ice to afford the crude product which was purified by column chromatography on silica gel. The pure compound was dissolved in ethanol to get single crystals.

### Refinement

An analysis of the E-statistics indicated the structure to be non-centrosymmetric. The centrosymmetric space group P -1 was attempted but it was not successful. However, the crystal was twinned and TWIN and BASF instructions were used in the refinement procedure. The H atoms were positioned geometrically with N—H = 0.86 Å, C—H = 0.93 and 0.96 Å, and refined a riding model, with *U*_iso_(H) = 1.2–1.5*U*_eq_(C). In the absence of significant anomalous dispersion effects, Freidel pairs were merged using the MERG 4 command in *SHELXL97*; Sheldrick, 2008).

### Crystal data

**Table:**

C_18_H_16_N_2_O	*Z* = 4
*M**_r_* = 276.33	*F*(000) = 584
Triclinic, *P*1	*D*_x_ = 1.288 Mg m^−^^3^
Hall symbol: P 1	Mo *K*α radiation, λ = 0.71073 Å
*a* = 9.7383 (5) Å	Cell parameters from 1432 reflections
*b* = 9.9035 (6) Å	θ = 2.0–20.5°
*c* = 15.2809 (9) Å	µ = 0.08 mm^−^^1^
α = 101.414 (5)°	*T* = 295 K
β = 99.188 (5)°	Block, colourless
γ = 90.065 (5)°	0.20 × 0.10 × 0.10 mm
*V* = 1425.24 (14) Å^3^	

### Data collection

**Table:**

Oxford Xcalibur Eos(Nova) CCD detector diffractometer	5290 independent reflections
Radiation source: Enhance (Mo) X-ray Source	3314 reflections with *I* > 2σ(*I*)
graphite	*R*_int_ = 0.079
ω scans	θ_max_ = 25.5°, θ_min_ = 2.7°
Absorption correction: multi-scan (*CrysAlis PRO* RED; Oxford Diffraction, 2009)	*h* = −11→11
*T*_min_ = 0.984, *T*_max_ = 0.992	*k* = −11→11
29328 measured reflections	*l* = −18→18

### Refinement

**Table:**

Refinement on *F*^2^	Primary atom site location: structure-invariant direct methods
Least-squares matrix: full	Secondary atom site location: difference Fourier map
*R*[*F*^2^ > 2σ(*F*^2^)] = 0.058	Hydrogen site location: inferred from neighbouring sites
*wR*(*F*^2^) = 0.164	H-atom parameters constrained
*S* = 0.94	*w* = 1/[σ^2^(*F*_o_^2^) + (0.1055*P*)^2^] where *P* = (*F*_o_^2^ + 2*F*_c_^2^)/3
5290 reflections	(Δ/σ)_max_ < 0.001
766 parameters	Δρ_max_ = 0.23 e Å^−^^3^
3 restraints	Δρ_min_ = −0.24 e Å^−^^3^

### Special details

**Table:**

Geometry. Bond distances, angles *etc*. have been calculated using the rounded fractional coordinates. All su's are estimated from the variances of the (full) variance-covariance matrix. The cell e.s.d.'s are taken into account in the estimation of distances, angles and torsion angles
Refinement. Refinement on *F*^2^ for ALL reflections except those flagged by the user for potential systematic errors. Weighted *R*-factors *wR* and all goodnesses of fit *S* are based on *F*^2^, conventional *R*-factors *R* are based on *F*, with *F* set to zero for negative *F*^2^. The observed criterion of *F*^2^ > σ(*F*^2^) is used only for calculating -*R*-factor-obs *etc*. and is not relevant to the choice of reflections for refinement. *R*-factors based on *F*^2^ are statistically about twice as large as those based on *F*, and *R*-factors based on ALL data will be even larger.

### Fractional atomic coordinates and isotropic or equivalent isotropic displacement parameters (Å^2^)

**Table:**

	*x*	*y*	*z*	*U*_iso_*/*U*_eq_	
O1	0.5433 (4)	0.3283 (5)	−0.0893 (3)	0.0679 (16)	
N1	0.9301 (5)	0.0670 (5)	0.0014 (3)	0.0477 (17)	
N2	0.7568 (5)	0.2789 (5)	−0.0233 (3)	0.0479 (17)	
C1	0.8509 (5)	0.0467 (6)	0.0608 (4)	0.0447 (19)	
C2	0.8302 (6)	−0.0875 (6)	0.0787 (4)	0.049 (2)	
C3	0.8889 (5)	−0.2001 (6)	0.0339 (4)	0.050 (2)	
C4	0.9720 (5)	−0.1799 (6)	−0.0317 (4)	0.0468 (19)	
C5	1.0340 (6)	−0.2906 (7)	−0.0856 (4)	0.058 (2)	
C6	1.1092 (7)	−0.2636 (7)	−0.1487 (4)	0.064 (3)	
C7	1.1259 (7)	−0.1275 (7)	−0.1600 (4)	0.063 (3)	
C8	1.0672 (6)	−0.0231 (7)	−0.1108 (4)	0.057 (2)	
C9	0.9890 (5)	−0.0465 (6)	−0.0459 (4)	0.047 (2)	
C10	0.8644 (7)	−0.3407 (7)	0.0521 (5)	0.066 (3)	
C11	0.7762 (6)	0.1633 (5)	0.1048 (4)	0.0446 (17)	
C12	0.7446 (7)	0.1650 (6)	0.1914 (4)	0.057 (2)	
C13	0.6635 (7)	0.2636 (8)	0.2323 (4)	0.065 (3)	
C14	0.6128 (7)	0.3667 (7)	0.1878 (4)	0.062 (2)	
C15	0.6434 (6)	0.3709 (6)	0.1040 (4)	0.0507 (19)	
C16	0.7258 (5)	0.2726 (6)	0.0623 (4)	0.0442 (17)	
C17	0.6659 (6)	0.3079 (6)	−0.0933 (4)	0.049 (2)	
C18	0.7213 (6)	0.3083 (8)	−0.1781 (4)	0.066 (2)	
O2	0.2107 (4)	0.0456 (6)	0.5805 (3)	0.0728 (18)	
N3	0.5519 (5)	0.3067 (5)	0.4898 (3)	0.0489 (17)	
N4	0.3917 (5)	0.0960 (5)	0.5163 (3)	0.0500 (17)	
C19	0.4453 (6)	0.3265 (6)	0.4306 (4)	0.0429 (17)	
C20	0.4127 (6)	0.4601 (6)	0.4125 (4)	0.051 (2)	
C21	0.4952 (6)	0.5736 (6)	0.4563 (4)	0.0477 (19)	
C22	0.6121 (5)	0.5530 (6)	0.5222 (4)	0.0435 (17)	
C23	0.7021 (7)	0.6627 (7)	0.5749 (4)	0.059 (2)	
C24	0.8104 (7)	0.6361 (7)	0.6365 (4)	0.063 (3)	
C25	0.8316 (6)	0.5019 (7)	0.6502 (4)	0.061 (3)	
C26	0.7462 (6)	0.3954 (7)	0.6017 (4)	0.057 (2)	
C27	0.6368 (6)	0.4191 (5)	0.5363 (4)	0.0437 (17)	
C28	0.4617 (7)	0.7137 (6)	0.4391 (5)	0.068 (3)	
C29	0.3475 (6)	0.2082 (6)	0.3856 (4)	0.0465 (19)	
C30	0.2745 (7)	0.2048 (7)	0.2995 (4)	0.061 (2)	
C31	0.1718 (7)	0.1061 (8)	0.2583 (5)	0.070 (3)	
C32	0.1437 (7)	0.0043 (7)	0.3041 (5)	0.065 (3)	
C33	0.2144 (6)	0.0003 (6)	0.3879 (4)	0.0527 (19)	
C34	0.3185 (5)	0.1019 (6)	0.4296 (4)	0.050 (2)	
C35	0.3340 (6)	0.0675 (6)	0.5860 (4)	0.051 (2)	
C36	0.4340 (6)	0.0684 (8)	0.6700 (4)	0.066 (3)	
O3	0.7086 (4)	1.0413 (5)	0.5781 (3)	0.0692 (16)	
N5	1.0455 (4)	0.7212 (5)	0.4866 (3)	0.0451 (16)	
N6	0.8859 (5)	0.9444 (5)	0.5108 (3)	0.0479 (16)	
C37	0.9378 (5)	0.6617 (6)	0.4268 (3)	0.0423 (17)	
C38	0.9110 (6)	0.5172 (6)	0.4096 (4)	0.0480 (17)	
C39	0.9949 (6)	0.4330 (6)	0.4534 (4)	0.050 (2)	
C40	1.1085 (6)	0.4948 (6)	0.5202 (4)	0.0474 (19)	
C41	1.1998 (7)	0.4191 (7)	0.5722 (4)	0.057 (2)	
C42	1.3077 (7)	0.4881 (8)	0.6362 (5)	0.064 (3)	
C43	1.3243 (6)	0.6285 (8)	0.6493 (4)	0.064 (3)	
C44	1.2377 (6)	0.7027 (7)	0.5996 (4)	0.054 (2)	
C45	1.1293 (5)	0.6383 (6)	0.5340 (4)	0.0441 (19)	
C46	0.9622 (7)	0.2814 (7)	0.4362 (5)	0.069 (3)	
C47	0.8399 (6)	0.7511 (6)	0.3819 (4)	0.0448 (17)	
C48	0.7663 (7)	0.7020 (7)	0.2959 (4)	0.057 (2)	
C49	0.6643 (7)	0.7741 (8)	0.2551 (4)	0.067 (3)	
C50	0.6357 (6)	0.9044 (7)	0.2999 (5)	0.061 (3)	
C51	0.7087 (6)	0.9602 (7)	0.3832 (4)	0.053 (2)	
C52	0.8113 (5)	0.8866 (6)	0.4248 (4)	0.0446 (17)	
C53	0.8323 (6)	1.0137 (6)	0.5815 (4)	0.050 (2)	
C54	0.9305 (7)	1.0539 (8)	0.6673 (4)	0.070 (3)	
O4	0.0423 (4)	0.3327 (6)	−0.0889 (3)	0.0733 (18)	
N7	0.4267 (5)	0.6515 (5)	0.0017 (3)	0.0443 (16)	
N8	0.2536 (5)	0.4286 (5)	−0.0231 (3)	0.0471 (16)	
C55	0.3477 (6)	0.7108 (6)	0.0610 (3)	0.0449 (17)	
C56	0.3282 (6)	0.8550 (6)	0.0788 (4)	0.049 (2)	
C57	0.3881 (6)	0.9399 (6)	0.0347 (4)	0.0529 (19)	
C58	0.4710 (5)	0.8799 (6)	−0.0307 (4)	0.0443 (19)	
C59	0.5375 (6)	0.9536 (7)	−0.0836 (5)	0.060 (2)	
C60	0.6132 (6)	0.8888 (7)	−0.1453 (4)	0.060 (3)	
C61	0.6281 (6)	0.7468 (8)	−0.1591 (4)	0.060 (2)	
C62	0.5646 (6)	0.6722 (7)	−0.1089 (4)	0.056 (2)	
C63	0.4868 (5)	0.7360 (6)	−0.0448 (4)	0.0447 (19)	
C64	0.3641 (7)	1.0908 (7)	0.0524 (5)	0.069 (3)	
C65	0.2726 (5)	0.6201 (5)	0.1057 (3)	0.0419 (17)	
C66	0.2424 (6)	0.6714 (7)	0.1922 (4)	0.058 (2)	
C67	0.1606 (7)	0.5957 (8)	0.2330 (4)	0.064 (3)	
C68	0.1105 (7)	0.4662 (7)	0.1882 (5)	0.062 (3)	
C69	0.1415 (6)	0.4109 (6)	0.1048 (4)	0.0527 (19)	
C70	0.2233 (5)	0.4852 (6)	0.0627 (4)	0.0461 (19)	
C71	0.1644 (6)	0.3579 (6)	−0.0941 (4)	0.050 (2)	
C72	0.2186 (7)	0.3182 (8)	−0.1798 (5)	0.067 (3)	
H2	0.77570	−0.09830	0.12170	0.0580*	
H2N	0.84080	0.26300	−0.03210	0.0580*	
H5	1.02300	−0.38050	−0.07780	0.0690*	
H6	1.14940	−0.33520	−0.18410	0.0770*	
H7	1.17860	−0.10960	−0.20240	0.0760*	
H8	1.07860	0.06600	−0.11990	0.0680*	
H10A	0.93300	−0.35620	0.10140	0.0990*	
H10B	0.87120	−0.40900	−0.00100	0.0990*	
H10C	0.77320	−0.34690	0.06770	0.0990*	
H12	0.77970	0.09710	0.22210	0.0680*	
H13	0.64290	0.26120	0.28930	0.0770*	
H14	0.55760	0.43360	0.21500	0.0750*	
H15	0.60850	0.44050	0.07480	0.0610*	
H18A	0.64540	0.30660	−0.22690	0.0980*	
H18B	0.77600	0.22850	−0.19150	0.0980*	
H18C	0.77850	0.39000	−0.17100	0.0980*	
H4N	0.48020	0.11190	0.52570	0.0600*	
H20	0.33480	0.47020	0.37070	0.0620*	
H23	0.68700	0.75260	0.56720	0.0710*	
H24	0.87060	0.70770	0.66970	0.0750*	
H25	0.90530	0.48520	0.69310	0.0730*	
H26	0.76060	0.30680	0.61210	0.0680*	
H28A	0.37030	0.71110	0.40410	0.1020*	
H28B	0.52860	0.74310	0.40630	0.1020*	
H28C	0.46460	0.77720	0.49570	0.1020*	
H30	0.29560	0.27170	0.26820	0.0730*	
H31	0.12250	0.10780	0.20120	0.0840*	
H32	0.07490	−0.06290	0.27700	0.0780*	
H33	0.19410	−0.06910	0.41740	0.0630*	
H36A	0.41260	0.14110	0.71710	0.0980*	
H36B	0.52680	0.08330	0.65930	0.0980*	
H36C	0.42730	−0.01850	0.68810	0.0980*	
H6N	0.97440	0.93430	0.51890	0.0570*	
H38	0.83430	0.47900	0.36750	0.0580*	
H41	1.18780	0.32400	0.56370	0.0690*	
H42	1.36880	0.43880	0.67010	0.0780*	
H43	1.39590	0.67370	0.69300	0.0770*	
H44	1.25110	0.79790	0.60960	0.0650*	
H46A	0.88630	0.25780	0.38680	0.1030*	
H46B	0.93660	0.25760	0.48950	0.1030*	
H46C	1.04260	0.23170	0.42120	0.1030*	
H48	0.78730	0.61610	0.26460	0.0690*	
H49	0.61470	0.73650	0.19830	0.0800*	
H50	0.56610	0.95420	0.27290	0.0740*	
H51	0.68940	1.04830	0.41210	0.0640*	
H54A	0.91000	1.14440	0.69750	0.1050*	
H54B	1.02400	1.05440	0.65470	0.1050*	
H54C	0.92150	0.98920	0.70530	0.1050*	
H8N	0.33780	0.44010	−0.03140	0.0560*	
H56	0.27320	0.89230	0.12150	0.0590*	
H59	0.52900	1.04860	−0.07570	0.0720*	
H60	0.65590	0.93980	−0.17910	0.0710*	
H61	0.68030	0.70290	−0.20170	0.0730*	
H62	0.57400	0.57730	−0.11810	0.0670*	
H64A	0.29360	1.11030	0.09000	0.1040*	
H64B	0.33430	1.11930	−0.00390	0.1040*	
H64C	0.44910	1.14000	0.08250	0.1040*	
H66	0.27770	0.75810	0.22310	0.0690*	
H67	0.13980	0.63210	0.29020	0.0770*	
H68	0.05490	0.41570	0.21510	0.0740*	
H69	0.10780	0.32280	0.07580	0.0630*	
H72A	0.24060	0.22250	−0.18900	0.1010*	
H72B	0.30100	0.37300	−0.17740	0.1010*	
H72C	0.14950	0.33340	−0.22890	0.1010*	

### Atomic displacement parameters (Å^2^)

**Table:**

	*U*^11^	*U*^22^	*U*^33^	*U*^12^	*U*^13^	*U*^23^
O1	0.037 (2)	0.086 (3)	0.087 (3)	0.012 (2)	0.013 (2)	0.030 (3)
N1	0.042 (3)	0.044 (3)	0.059 (3)	−0.001 (2)	0.009 (2)	0.014 (2)
N2	0.041 (3)	0.050 (3)	0.056 (3)	0.000 (2)	0.010 (2)	0.017 (2)
C1	0.034 (3)	0.053 (4)	0.045 (3)	−0.003 (3)	−0.001 (2)	0.011 (3)
C2	0.040 (3)	0.050 (4)	0.059 (4)	0.003 (3)	0.007 (3)	0.019 (3)
C3	0.036 (3)	0.051 (4)	0.062 (4)	0.001 (3)	−0.003 (3)	0.021 (3)
C4	0.033 (3)	0.054 (4)	0.050 (3)	0.008 (3)	−0.003 (2)	0.010 (3)
C5	0.050 (4)	0.049 (4)	0.069 (4)	0.010 (3)	−0.007 (3)	0.014 (3)
C6	0.064 (4)	0.062 (5)	0.065 (4)	0.027 (3)	0.014 (3)	0.007 (3)
C7	0.052 (4)	0.071 (5)	0.067 (4)	0.007 (3)	0.014 (3)	0.015 (4)
C8	0.050 (4)	0.063 (4)	0.061 (4)	0.002 (3)	0.011 (3)	0.016 (3)
C9	0.033 (3)	0.050 (4)	0.055 (4)	0.007 (3)	−0.005 (3)	0.012 (3)
C10	0.059 (4)	0.055 (4)	0.091 (5)	0.002 (3)	0.009 (4)	0.034 (4)
C11	0.044 (3)	0.038 (3)	0.050 (3)	0.001 (2)	0.006 (3)	0.006 (3)
C12	0.063 (4)	0.046 (4)	0.059 (4)	−0.004 (3)	0.007 (3)	0.009 (3)
C13	0.067 (4)	0.075 (5)	0.051 (4)	−0.010 (4)	0.022 (3)	0.001 (3)
C14	0.060 (4)	0.056 (4)	0.067 (4)	−0.005 (3)	0.025 (3)	−0.008 (3)
C15	0.044 (3)	0.036 (3)	0.072 (4)	−0.001 (3)	0.014 (3)	0.007 (3)
C16	0.036 (3)	0.038 (3)	0.057 (3)	−0.006 (2)	0.007 (2)	0.006 (3)
C17	0.046 (4)	0.045 (3)	0.059 (4)	−0.003 (3)	0.006 (3)	0.017 (3)
C18	0.047 (3)	0.091 (5)	0.061 (4)	0.011 (3)	0.011 (3)	0.017 (3)
O2	0.038 (2)	0.097 (4)	0.090 (3)	−0.011 (2)	0.015 (2)	0.031 (3)
N3	0.043 (3)	0.052 (3)	0.056 (3)	−0.001 (2)	0.011 (2)	0.019 (2)
N4	0.039 (3)	0.048 (3)	0.067 (3)	0.004 (2)	0.013 (2)	0.018 (2)
C19	0.042 (3)	0.045 (3)	0.047 (3)	0.002 (3)	0.018 (3)	0.013 (3)
C20	0.051 (3)	0.051 (4)	0.058 (4)	0.000 (3)	0.015 (3)	0.021 (3)
C21	0.048 (3)	0.040 (3)	0.064 (4)	0.001 (3)	0.025 (3)	0.019 (3)
C22	0.040 (3)	0.047 (3)	0.050 (3)	−0.003 (3)	0.020 (3)	0.015 (3)
C23	0.056 (4)	0.049 (4)	0.079 (4)	−0.003 (3)	0.027 (4)	0.018 (3)
C24	0.056 (4)	0.069 (5)	0.064 (4)	−0.022 (3)	0.014 (3)	0.011 (3)
C25	0.052 (4)	0.072 (5)	0.059 (4)	−0.012 (3)	0.005 (3)	0.014 (3)
C26	0.045 (3)	0.059 (4)	0.072 (4)	0.002 (3)	0.017 (3)	0.022 (3)
C27	0.044 (3)	0.040 (3)	0.052 (3)	−0.005 (3)	0.020 (3)	0.011 (3)
C28	0.070 (4)	0.048 (4)	0.095 (5)	−0.001 (3)	0.020 (4)	0.030 (4)
C29	0.041 (3)	0.046 (3)	0.054 (4)	0.011 (3)	0.012 (3)	0.010 (3)
C30	0.063 (4)	0.059 (4)	0.061 (4)	0.000 (3)	0.011 (3)	0.010 (3)
C31	0.062 (4)	0.071 (5)	0.064 (4)	0.005 (4)	−0.006 (3)	−0.003 (4)
C32	0.060 (4)	0.051 (4)	0.077 (5)	0.005 (3)	0.006 (4)	0.000 (3)
C33	0.042 (3)	0.040 (3)	0.074 (4)	0.005 (3)	0.010 (3)	0.006 (3)
C34	0.037 (3)	0.051 (4)	0.059 (4)	0.011 (3)	0.008 (3)	0.007 (3)
C35	0.046 (4)	0.037 (3)	0.074 (4)	0.002 (3)	0.013 (3)	0.018 (3)
C36	0.051 (4)	0.086 (5)	0.063 (4)	−0.006 (3)	0.010 (3)	0.022 (4)
O3	0.036 (2)	0.081 (3)	0.087 (3)	0.014 (2)	0.014 (2)	0.005 (2)
N5	0.039 (2)	0.043 (3)	0.055 (3)	0.001 (2)	0.013 (2)	0.010 (2)
N6	0.035 (2)	0.047 (3)	0.060 (3)	0.002 (2)	0.006 (2)	0.008 (2)
C37	0.040 (3)	0.046 (3)	0.042 (3)	−0.001 (3)	0.011 (2)	0.008 (2)
C38	0.043 (3)	0.046 (3)	0.052 (3)	0.000 (3)	0.012 (3)	−0.001 (3)
C39	0.054 (4)	0.041 (3)	0.058 (4)	0.009 (3)	0.020 (3)	0.007 (3)
C40	0.043 (3)	0.053 (4)	0.052 (3)	0.013 (3)	0.021 (3)	0.014 (3)
C41	0.059 (4)	0.048 (4)	0.074 (4)	0.013 (3)	0.029 (3)	0.019 (3)
C42	0.051 (4)	0.079 (5)	0.072 (4)	0.016 (4)	0.012 (3)	0.035 (4)
C43	0.043 (3)	0.086 (6)	0.064 (4)	0.000 (3)	0.008 (3)	0.020 (4)
C44	0.043 (3)	0.053 (4)	0.068 (4)	0.005 (3)	0.009 (3)	0.014 (3)
C45	0.036 (3)	0.047 (4)	0.054 (3)	0.007 (3)	0.017 (3)	0.014 (3)
C46	0.075 (5)	0.045 (4)	0.085 (5)	0.002 (3)	0.015 (4)	0.010 (3)
C47	0.041 (3)	0.046 (3)	0.050 (3)	−0.001 (3)	0.009 (3)	0.015 (3)
C48	0.062 (4)	0.056 (4)	0.055 (4)	−0.001 (3)	0.010 (3)	0.015 (3)
C49	0.060 (4)	0.081 (5)	0.059 (4)	−0.015 (4)	−0.010 (3)	0.028 (4)
C50	0.052 (4)	0.064 (5)	0.075 (4)	−0.002 (3)	0.001 (3)	0.038 (4)
C51	0.047 (3)	0.055 (4)	0.065 (4)	0.002 (3)	0.007 (3)	0.030 (3)
C52	0.042 (3)	0.040 (3)	0.054 (3)	−0.006 (3)	0.011 (3)	0.012 (3)
C53	0.047 (4)	0.040 (3)	0.063 (4)	−0.001 (3)	0.015 (3)	0.009 (3)
C54	0.054 (4)	0.088 (5)	0.061 (4)	0.013 (4)	0.011 (3)	−0.004 (4)
O4	0.037 (2)	0.096 (4)	0.085 (3)	−0.015 (2)	0.013 (2)	0.011 (3)
N7	0.040 (2)	0.039 (3)	0.054 (3)	0.001 (2)	0.007 (2)	0.010 (2)
N8	0.041 (2)	0.042 (3)	0.059 (3)	−0.003 (2)	0.014 (2)	0.007 (2)
C55	0.042 (3)	0.047 (3)	0.045 (3)	0.003 (3)	0.007 (3)	0.008 (3)
C56	0.045 (3)	0.046 (4)	0.057 (4)	−0.005 (3)	0.011 (3)	0.008 (3)
C57	0.046 (3)	0.043 (3)	0.066 (4)	−0.008 (3)	0.001 (3)	0.009 (3)
C58	0.032 (3)	0.049 (4)	0.048 (3)	−0.010 (2)	−0.005 (2)	0.010 (3)
C59	0.049 (3)	0.053 (4)	0.076 (4)	−0.018 (3)	−0.003 (3)	0.022 (3)
C60	0.052 (4)	0.065 (5)	0.066 (4)	−0.013 (3)	0.006 (3)	0.026 (3)
C61	0.040 (3)	0.085 (5)	0.061 (4)	0.002 (3)	0.014 (3)	0.022 (4)
C62	0.045 (3)	0.061 (4)	0.062 (4)	0.004 (3)	0.007 (3)	0.014 (3)
C63	0.032 (3)	0.045 (4)	0.056 (3)	−0.004 (3)	0.000 (3)	0.013 (3)
C64	0.059 (4)	0.051 (4)	0.097 (5)	−0.010 (3)	0.014 (4)	0.012 (4)
C65	0.038 (3)	0.041 (3)	0.047 (3)	−0.003 (2)	0.008 (2)	0.009 (3)
C66	0.058 (4)	0.056 (4)	0.060 (4)	−0.002 (3)	0.014 (3)	0.011 (3)
C67	0.066 (4)	0.083 (5)	0.052 (4)	0.016 (4)	0.021 (3)	0.027 (4)
C68	0.059 (4)	0.064 (5)	0.074 (4)	0.009 (3)	0.023 (3)	0.033 (4)
C69	0.049 (3)	0.040 (3)	0.075 (4)	0.003 (3)	0.016 (3)	0.021 (3)
C70	0.041 (3)	0.041 (3)	0.058 (4)	0.003 (3)	0.007 (3)	0.015 (3)
C71	0.040 (3)	0.046 (4)	0.062 (4)	0.000 (3)	0.006 (3)	0.008 (3)
C72	0.047 (4)	0.076 (5)	0.074 (4)	−0.007 (3)	0.010 (3)	0.003 (3)

### Geometric parameters (Å, °)

**Table:**

O1—C17	1.220 (7)	C25—H25	0.9300
O2—C35	1.207 (7)	C26—H26	0.9300
O3—C53	1.231 (7)	C28—H28A	0.9600
O4—C71	1.233 (7)	C28—H28B	0.9600
N1—C9	1.388 (8)	C28—H28C	0.9600
N1—C1	1.325 (7)	C30—H30	0.9300
N2—C17	1.353 (8)	C31—H31	0.9300
N2—C16	1.402 (7)	C32—H32	0.9300
N2—H2N	0.8600	C33—H33	0.9300
N3—C19	1.306 (8)	C36—H36B	0.9600
N3—C27	1.387 (7)	C36—H36A	0.9600
N4—C35	1.356 (8)	C36—H36C	0.9600
N4—C34	1.413 (7)	C37—C47	1.482 (8)
N4—H4N	0.8600	C37—C38	1.419 (8)
N5—C45	1.383 (7)	C38—C39	1.363 (8)
N5—C37	1.327 (6)	C39—C46	1.497 (9)
N6—C53	1.341 (8)	C39—C40	1.425 (8)
N6—C52	1.407 (7)	C40—C45	1.404 (9)
N6—H6N	0.8600	C40—C41	1.413 (9)
N7—C63	1.384 (8)	C41—C42	1.390 (10)
N7—C55	1.331 (7)	C42—C43	1.371 (11)
N8—C71	1.349 (8)	C43—C44	1.359 (9)
N8—C70	1.398 (7)	C44—C45	1.391 (8)
N8—H8N	0.8600	C47—C48	1.387 (8)
C1—C11	1.472 (8)	C47—C52	1.421 (8)
C1—C2	1.429 (9)	C48—C49	1.366 (10)
C2—C3	1.365 (8)	C49—C50	1.387 (10)
C3—C10	1.499 (9)	C50—C51	1.365 (9)
C3—C4	1.428 (8)	C51—C52	1.384 (8)
C4—C5	1.431 (9)	C53—C54	1.478 (9)
C4—C9	1.394 (9)	C38—H38	0.9300
C5—C6	1.366 (9)	C41—H41	0.9300
C6—C7	1.405 (10)	C42—H42	0.9300
C7—C8	1.339 (9)	C43—H43	0.9300
C8—C9	1.396 (8)	C44—H44	0.9300
C11—C12	1.403 (8)	C46—H46B	0.9600
C11—C16	1.419 (8)	C46—H46A	0.9600
C12—C13	1.373 (10)	C46—H46C	0.9600
C13—C14	1.385 (10)	C48—H48	0.9300
C14—C15	1.369 (9)	C49—H49	0.9300
C15—C16	1.385 (8)	C50—H50	0.9300
C17—C18	1.482 (8)	C51—H51	0.9300
C2—H2	0.9300	C54—H54B	0.9600
C5—H5	0.9300	C54—H54A	0.9600
C6—H6	0.9300	C54—H54C	0.9600
C7—H7	0.9300	C55—C65	1.486 (8)
C8—H8	0.9300	C55—C56	1.419 (8)
C10—H10A	0.9600	C56—C57	1.360 (8)
C10—H10C	0.9600	C57—C64	1.491 (9)
C10—H10B	0.9600	C57—C58	1.424 (8)
C12—H12	0.9300	C58—C63	1.411 (9)
C13—H13	0.9300	C58—C59	1.416 (9)
C14—H14	0.9300	C59—C60	1.352 (9)
C15—H15	0.9300	C60—C61	1.391 (11)
C18—H18A	0.9600	C61—C62	1.375 (9)
C18—H18B	0.9600	C62—C63	1.388 (8)
C18—H18C	0.9600	C65—C70	1.413 (8)
C19—C20	1.430 (9)	C65—C66	1.397 (7)
C19—C29	1.491 (8)	C66—C67	1.388 (10)
C20—C21	1.373 (8)	C67—C68	1.377 (10)
C21—C22	1.438 (8)	C68—C69	1.365 (9)
C21—C28	1.490 (9)	C69—C70	1.393 (8)
C22—C23	1.422 (9)	C71—C72	1.472 (9)
C22—C27	1.400 (8)	C56—H56	0.9300
C23—C24	1.360 (9)	C59—H59	0.9300
C24—C25	1.396 (10)	C60—H60	0.9300
C25—C26	1.358 (9)	C61—H61	0.9300
C26—C27	1.394 (8)	C62—H62	0.9300
C29—C34	1.407 (8)	C64—H64A	0.9600
C29—C30	1.386 (8)	C64—H64B	0.9600
C30—C31	1.380 (10)	C64—H64C	0.9600
C31—C32	1.386 (11)	C66—H66	0.9300
C32—C33	1.362 (9)	C67—H67	0.9300
C33—C34	1.409 (8)	C68—H68	0.9300
C35—C36	1.481 (8)	C69—H69	0.9300
C20—H20	0.9300	C72—H72A	0.9600
C23—H23	0.9300	C72—H72B	0.9600
C24—H24	0.9300	C72—H72C	0.9600
			
O1···N8	3.239 (6)	C59···H64B	2.8400
O1···C15	2.905 (7)	C59···H64C	3.0700
O1···C72	3.236 (8)	C61···H43^xii^	2.9100
O2···N6^i^	3.262 (6)	C64···H59	2.7000
O2···C33	2.894 (7)	C66···H56	2.6600
O2···C54^i^	3.213 (8)	C66···H10A^v^	3.1000
O3···C51	2.927 (7)	C67···H20	2.9600
O3···C36^ii^	3.201 (7)	C67···H28A	3.0700
O3···N4^ii^	3.160 (6)	C67···H10A^v^	2.8500
O4···C18^iii^	3.193 (7)	C68···H30	2.9500
O4···N2^iii^	3.181 (6)	C68···H10A^v^	2.8400
O4···C69	2.911 (7)	C69···H10A^v^	3.0800
O1···H8N	2.4900	C71···H69	2.8200
O1···H15	2.5100	C71···H8^iii^	2.9400
O1···H72B	2.6100	C71···H5^v^	2.9200
O1···H62	2.6100	C72···H36A^xiii^	3.0000
O1···H59^iv^	2.8200	H2···C12	2.6500
O2···H54B^i^	2.2800	H2···H12	2.2100
O2···H33	2.5100	H2···C49^iv^	2.9400
O2···H44^i^	2.6000	H2···H10C	2.4400
O2···H6N^i^	2.5100	H2N···O4^vi^	2.4200
O2···H41^iii^	2.8300	H2N···C1	2.7900
O3···H51	2.5300	H2N···N1	2.2500
O3···H26^ii^	2.6100	H2N···H18B	2.3700
O3···H4N^ii^	2.3900	H4N···H36B	2.0900
O3···H36B^ii^	2.3100	H4N···C19	2.7900
O3···H23	2.8400	H4N···O3^iv^	2.3900
O4···H8^iii^	2.6200	H4N···N3	2.2500
O4···H5^v^	2.8200	H5···C71^viii^	2.9200
O4···H69	2.5200	H5···H10B	2.0800
O4···H2N^iii^	2.4200	H5···C10	2.6700
O4···H18C^iii^	2.7800	H5···O4^viii^	2.8200
O4···H18B^iii^	2.8800	H6N···C37	2.7800
N1···N2	2.741 (7)	H6N···O2^vii^	2.5100
N2···O4^vi^	3.181 (6)	H6N···N5	2.2100
N2···N1	2.741 (7)	H6N···H54B	2.1600
N3···N4	2.735 (7)	H8···O4^vi^	2.6200
N4···O3^iv^	3.160 (6)	H8···C71^vi^	2.9400
N4···N3	2.735 (7)	H8N···O1	2.4900
N5···N6	2.703 (7)	H8N···N7	2.2000
N6···O2^vii^	3.262 (6)	H8N···H72B	2.1700
N6···N5	2.703 (7)	H8N···C55	2.7700
N7···N8	2.705 (7)	H10A···C69^viii^	3.0800
N8···O1	3.239 (6)	H10A···C66^viii^	3.1000
N8···N7	2.705 (7)	H10A···C67^viii^	2.8500
N1···H2N	2.2500	H10A···C68^viii^	2.8400
N3···H4N	2.2500	H10B···C5	2.6100
N5···H6N	2.2100	H10B···H5	2.0800
N7···H8N	2.2000	H10C···H2	2.4400
C1···C59^iv^	3.474 (8)	H12···C50^iv^	2.9100
C2···C59^iv^	3.548 (9)	H12···H2	2.2100
C3···C61^iv^	3.524 (8)	H12···C2	2.6700
C4···C61^iv^	3.590 (8)	H13···C19	3.0900
C4···C56^viii^	3.592 (8)	H13···H46A	2.5900
C5···C56^viii^	3.586 (8)	H15···C17	2.7900
C5···C55^viii^	3.486 (8)	H15···O1	2.5100
C7···C57^viii^	3.550 (9)	H18B···C54^ix^	3.0800
C8···C64^viii^	3.525 (9)	H18B···H2N	2.3700
C8···C57^viii^	3.599 (8)	H18B···H54A^ix^	2.3300
C10···C62^iv^	3.525 (9)	H18B···O4^vi^	2.8800
C12···C50^iv^	3.575 (9)	H18C···O4^vi^	2.7800
C15···O1	2.905 (7)	H20···H30	2.2500
C18···O4^vi^	3.193 (7)	H20···C30	2.6600
C19···C41^iii^	3.480 (9)	H20···H28A	2.3500
C20···C40^iii^	3.597 (8)	H20···C67	2.9600
C20···C41^iii^	3.525 (9)	H23···O3	2.8400
C21···C43^iii^	3.563 (8)	H23···H28C	2.3000
C22···C38	3.594 (8)	H23···C28	2.6700
C23···C38	3.578 (9)	H23···C53	2.9000
C23···C37	3.470 (8)	H26···H54A^iv^	2.5800
C25···C40	3.589 (8)	H26···O3^iv^	2.6100
C25···C39	3.572 (8)	H26···C53^iv^	2.9500
C26···C46	3.559 (9)	H28A···C67	3.0700
C28···C44^iii^	3.550 (9)	H28A···H20	2.3500
C33···O2	2.894 (7)	H28B···C52	3.0400
C36···O3^iv^	3.201 (7)	H28B···C23	3.0900
C37···C23	3.470 (8)	H28B···C48	3.0600
C38···C23	3.578 (9)	H28B···C49	2.9000
C38···C22	3.594 (8)	H28B···C50	2.8000
C39···C25	3.572 (8)	H28B···C51	2.8800
C40···C20^vi^	3.597 (8)	H28C···H23	2.3000
C40···C25	3.589 (8)	H28C···C44^iii^	3.0900
C41···C20^vi^	3.525 (9)	H28C···C23	2.7800
C41···C19^vi^	3.480 (9)	H30···C68	2.9500
C43···C21^vi^	3.563 (8)	H30···H20	2.2500
C44···C28^vi^	3.550 (9)	H30···C20	2.6800
C46···C26	3.559 (9)	H31···H64A^iv^	2.5700
C50···C12^ii^	3.575 (9)	H33···C35	2.8000
C51···O3	2.927 (7)	H33···O2	2.5100
C54···O2^vii^	3.213 (8)	H36A···H72A^x^	2.4100
C55···C5^v^	3.486 (8)	H36A···C72^x^	3.0000
C56···C5^v^	3.586 (8)	H36B···O3^iv^	2.3100
C56···C4^v^	3.592 (8)	H36B···H4N	2.0900
C57···C7^v^	3.550 (9)	H36C···H44^i^	2.4800
C57···C8^v^	3.599 (8)	H38···H46A	2.3100
C59···C2^ii^	3.548 (9)	H38···H48	2.2700
C59···C1^ii^	3.474 (8)	H38···C13	2.9600
C61···C4^ii^	3.590 (8)	H38···C48	2.6900
C61···C3^ii^	3.524 (8)	H41···C46	2.6700
C62···C10^ii^	3.525 (9)	H41···O2^vi^	2.8300
C64···C8^v^	3.525 (9)	H41···C35^vi^	2.9700
C69···O4	2.911 (7)	H41···H46C	2.4100
C72···O1	3.236 (8)	H41···H46B	2.5500
C1···H2N	2.7900	H43···C61^xiv^	2.9100
C2···H12	2.6700	H44···O2^vii^	2.6000
C5···H10B	2.6100	H44···C35^vii^	2.8900
C7···H54C^ix^	3.0300	H44···H36C^vii^	2.4800
C8···H64B^viii^	3.0200	H46A···C12	3.0400
C8···H54C^ix^	2.9600	H46A···C13	2.9500
C10···H5	2.6700	H46A···H38	2.3100
C12···H46A	3.0400	H46A···H13	2.5900
C12···H64C^iv^	3.0700	H46B···C26	2.8900
C12···H2	2.6500	H46B···C41	2.9800
C13···H38	2.9600	H46B···H41	2.5500
C13···H46A	2.9500	H46C···C31^vi^	3.0200
C13···H64C^iv^	2.9100	H46C···C32^vi^	2.8700
C14···H48	2.9300	H46C···H41	2.4100
C14···H64C^iv^	2.8100	H46C···C41	2.8800
C15···H64C^iv^	2.9000	H46C···C34^vi^	2.9800
C16···H64C^iv^	3.0800	H46C···C33^vi^	2.8500
C17···H62	2.8900	H48···H38	2.2700
C17···H15	2.7900	H48···C38	2.7100
C17···H59^iv^	2.9700	H48···C14	2.9300
C18···H62	3.0800	H49···C55	3.0500
C18···H54A^ix^	3.0900	H51···O3	2.5300
C19···H13	3.0900	H51···C53	2.8200
C19···H4N	2.7900	H54A···H26^ii^	2.5800
C20···H30	2.6800	H54A···C18^xi^	3.0900
C23···H28C	2.7800	H54A···H18B^xi^	2.3300
C23···H28B	3.0900	H54B···O2^vii^	2.2800
C24···H61^x^	2.9200	H54B···H6N	2.1600
C26···H46B	2.8900	H54C···C7^xi^	3.0300
C28···H23	2.6700	H54C···C8^xi^	2.9600
C30···H20	2.6600	H56···C31^ii^	2.9600
C31···H64A^iv^	3.0100	H56···C66	2.6600
C31···H46C^iii^	3.0200	H56···H64A	2.3200
C31···H56^iv^	2.9600	H56···H66	2.2300
C32···H66^iv^	2.9200	H59···O1^ii^	2.8200
C32···H46C^iii^	2.8700	H59···C17^ii^	2.9700
C33···H46C^iii^	2.8500	H59···C64	2.7000
C34···H46C^iii^	2.9800	H59···H64B	2.3800
C35···H33	2.8000	H61···C24^xiii^	2.9200
C35···H41^iii^	2.9700	H62···O1	2.6100
C35···H44^i^	2.8900	H62···C17	2.8900
C37···H6N	2.7800	H62···C18	3.0800
C37···H67^vi^	3.0600	H64A···C31^ii^	3.0100
C38···H48	2.7100	H64A···H31^ii^	2.5700
C41···H46B	2.9800	H64A···H56	2.3200
C41···H46C	2.8800	H64B···C8^v^	3.0200
C44···H28C^vi^	3.0900	H64B···C59	2.8400
C46···H41	2.6700	H64B···H59	2.3800
C48···H28B	3.0600	H64C···C12^ii^	3.0700
C48···H38	2.6900	H64C···C13^ii^	2.9100
C49···H2^ii^	2.9400	H64C···C14^ii^	2.8100
C49···H28B	2.9000	H64C···C15^ii^	2.9000
C50···H12^ii^	2.9100	H64C···C16^ii^	3.0800
C50···H28B	2.8000	H64C···C59	3.0700
C51···H28B	2.8800	H66···C32^ii^	2.9200
C52···H28B	3.0400	H66···C56	2.6800
C53···H26^ii^	2.9500	H66···H56	2.2300
C53···H23	2.9000	H67···C37^iii^	3.0600
C53···H51	2.8200	H69···O4	2.5200
C54···H18B^xi^	3.0800	H69···C71	2.8200
C55···H49	3.0500	H72A···H36A^xiii^	2.4100
C55···H8N	2.7700	H72B···O1	2.6100
C56···H66	2.6800	H72B···H8N	2.1700
			
C1—N1—C9	118.3 (5)	C32—C33—H33	120.00
C16—N2—C17	125.6 (5)	C34—C33—H33	120.00
C17—N2—H2N	117.00	H36B—C36—H36C	110.00
C16—N2—H2N	117.00	C35—C36—H36B	109.00
C19—N3—C27	118.8 (5)	C35—C36—H36A	109.00
C34—N4—C35	125.4 (5)	H36A—C36—H36C	109.00
C34—N4—H4N	117.00	C35—C36—H36C	109.00
C35—N4—H4N	117.00	H36A—C36—H36B	109.00
C37—N5—C45	117.9 (5)	N5—C37—C38	121.5 (5)
C52—N6—C53	126.3 (5)	N5—C37—C47	118.3 (5)
C53—N6—H6N	117.00	C38—C37—C47	120.1 (5)
C52—N6—H6N	117.00	C37—C38—C39	121.6 (5)
C55—N7—C63	117.2 (5)	C38—C39—C46	120.7 (6)
C70—N8—C71	126.7 (5)	C40—C39—C46	121.2 (5)
C71—N8—H8N	117.00	C38—C39—C40	118.0 (6)
C70—N8—H8N	117.00	C39—C40—C41	123.3 (6)
N1—C1—C2	121.3 (5)	C41—C40—C45	119.2 (5)
N1—C1—C11	119.1 (5)	C39—C40—C45	117.5 (5)
C2—C1—C11	119.6 (5)	C40—C41—C42	119.4 (6)
C1—C2—C3	121.3 (5)	C41—C42—C43	120.2 (7)
C4—C3—C10	121.3 (5)	C42—C43—C44	121.1 (6)
C2—C3—C4	117.9 (5)	C43—C44—C45	120.9 (6)
C2—C3—C10	120.8 (5)	N5—C45—C40	123.4 (5)
C3—C4—C9	118.2 (5)	N5—C45—C44	117.3 (5)
C5—C4—C9	118.7 (5)	C40—C45—C44	119.2 (6)
C3—C4—C5	123.1 (6)	C48—C47—C52	116.7 (6)
C4—C5—C6	119.7 (6)	C37—C47—C48	120.4 (5)
C5—C6—C7	119.9 (6)	C37—C47—C52	122.8 (5)
C6—C7—C8	121.1 (6)	C47—C48—C49	123.0 (6)
C7—C8—C9	120.8 (6)	C48—C49—C50	118.9 (6)
C4—C9—C8	119.8 (5)	C49—C50—C51	120.6 (6)
N1—C9—C8	117.2 (5)	C50—C51—C52	120.4 (6)
N1—C9—C4	123.0 (5)	N6—C52—C47	119.5 (5)
C1—C11—C12	119.5 (5)	N6—C52—C51	120.2 (5)
C1—C11—C16	123.6 (5)	C47—C52—C51	120.3 (5)
C12—C11—C16	116.9 (5)	N6—C53—C54	116.1 (5)
C11—C12—C13	122.2 (6)	O3—C53—C54	120.3 (6)
C12—C13—C14	119.4 (6)	O3—C53—N6	123.6 (5)
C13—C14—C15	120.5 (6)	C37—C38—H38	119.00
C14—C15—C16	120.7 (6)	C39—C38—H38	119.00
N2—C16—C15	120.0 (5)	C40—C41—H41	120.00
N2—C16—C11	119.7 (5)	C42—C41—H41	120.00
C11—C16—C15	120.3 (5)	C43—C42—H42	120.00
O1—C17—C18	120.7 (5)	C41—C42—H42	120.00
O1—C17—N2	122.8 (5)	C42—C43—H43	119.00
N2—C17—C18	116.5 (5)	C44—C43—H43	119.00
C3—C2—H2	119.00	C43—C44—H44	120.00
C1—C2—H2	119.00	C45—C44—H44	120.00
C4—C5—H5	120.00	H46A—C46—H46C	109.00
C6—C5—H5	120.00	C39—C46—H46C	109.00
C7—C6—H6	120.00	H46A—C46—H46B	109.00
C5—C6—H6	120.00	C39—C46—H46A	109.00
C6—C7—H7	119.00	C39—C46—H46B	109.00
C8—C7—H7	119.00	H46B—C46—H46C	110.00
C9—C8—H8	120.00	C49—C48—H48	119.00
C7—C8—H8	120.00	C47—C48—H48	119.00
C3—C10—H10A	109.00	C48—C49—H49	121.00
H10B—C10—H10C	109.00	C50—C49—H49	120.00
H10A—C10—H10B	110.00	C51—C50—H50	120.00
C3—C10—H10B	110.00	C49—C50—H50	120.00
C3—C10—H10C	109.00	C50—C51—H51	120.00
H10A—C10—H10C	109.00	C52—C51—H51	120.00
C11—C12—H12	119.00	H54A—C54—H54C	110.00
C13—C12—H12	119.00	H54B—C54—H54C	109.00
C14—C13—H13	120.00	H54A—C54—H54B	109.00
C12—C13—H13	120.00	C53—C54—H54A	109.00
C15—C14—H14	120.00	C53—C54—H54B	109.00
C13—C14—H14	120.00	C53—C54—H54C	110.00
C16—C15—H15	120.00	C56—C55—C65	119.8 (5)
C14—C15—H15	120.00	N7—C55—C56	122.2 (5)
C17—C18—H18B	110.00	N7—C55—C65	117.9 (5)
C17—C18—H18A	110.00	C55—C56—C57	121.4 (5)
H18A—C18—H18B	109.00	C56—C57—C58	118.1 (6)
H18A—C18—H18C	109.00	C56—C57—C64	120.9 (6)
C17—C18—H18C	110.00	C58—C57—C64	121.0 (6)
H18B—C18—H18C	109.00	C57—C58—C59	124.9 (6)
N3—C19—C20	122.3 (5)	C59—C58—C63	117.4 (5)
N3—C19—C29	119.1 (5)	C57—C58—C63	117.7 (5)
C20—C19—C29	118.5 (5)	C58—C59—C60	121.4 (6)
C19—C20—C21	120.7 (5)	C59—C60—C61	120.9 (6)
C20—C21—C22	117.5 (5)	C60—C61—C62	119.1 (6)
C20—C21—C28	121.4 (6)	C61—C62—C63	121.2 (6)
C22—C21—C28	121.2 (5)	N7—C63—C58	123.4 (5)
C21—C22—C23	123.0 (6)	C58—C63—C62	119.9 (5)
C21—C22—C27	118.4 (5)	N7—C63—C62	116.7 (6)
C23—C22—C27	118.5 (5)	C55—C65—C70	122.9 (4)
C22—C23—C24	120.0 (6)	C66—C65—C70	117.8 (5)
C23—C24—C25	120.3 (6)	C55—C65—C66	119.3 (5)
C24—C25—C26	120.9 (6)	C65—C66—C67	121.3 (6)
C25—C26—C27	120.0 (6)	C66—C67—C68	119.5 (6)
N3—C27—C22	122.4 (5)	C67—C68—C69	120.9 (6)
N3—C27—C26	117.4 (5)	C68—C69—C70	120.6 (6)
C22—C27—C26	120.2 (5)	N8—C70—C69	120.3 (5)
C30—C29—C34	117.5 (6)	C65—C70—C69	119.9 (5)
C19—C29—C34	122.4 (5)	N8—C70—C65	119.7 (5)
C19—C29—C30	120.0 (6)	N8—C71—C72	116.4 (5)
C29—C30—C31	122.6 (6)	O4—C71—N8	122.1 (5)
C30—C31—C32	118.4 (7)	O4—C71—C72	121.5 (6)
C31—C32—C33	121.6 (6)	C55—C56—H56	119.00
C32—C33—C34	119.6 (6)	C57—C56—H56	119.00
N4—C34—C29	120.8 (5)	C58—C59—H59	119.00
N4—C34—C33	119.0 (5)	C60—C59—H59	119.00
C29—C34—C33	120.2 (5)	C59—C60—H60	120.00
N4—C35—C36	114.6 (5)	C61—C60—H60	119.00
O2—C35—N4	122.7 (6)	C60—C61—H61	120.00
O2—C35—C36	122.7 (6)	C62—C61—H61	120.00
C21—C20—H20	120.00	C61—C62—H62	119.00
C19—C20—H20	120.00	C63—C62—H62	119.00
C22—C23—H23	120.00	C57—C64—H64A	109.00
C24—C23—H23	120.00	C57—C64—H64B	109.00
C25—C24—H24	120.00	C57—C64—H64C	109.00
C23—C24—H24	120.00	H64A—C64—H64B	109.00
C26—C25—H25	120.00	H64A—C64—H64C	110.00
C24—C25—H25	120.00	H64B—C64—H64C	109.00
C25—C26—H26	120.00	C65—C66—H66	119.00
C27—C26—H26	120.00	C67—C66—H66	119.00
H28A—C28—H28C	109.00	C66—C67—H67	120.00
C21—C28—H28A	109.00	C68—C67—H67	120.00
H28A—C28—H28B	109.00	C67—C68—H68	120.00
C21—C28—H28B	109.00	C69—C68—H68	120.00
H28B—C28—H28C	109.00	C68—C69—H69	120.00
C21—C28—H28C	110.00	C70—C69—H69	120.00
C29—C30—H30	119.00	C71—C72—H72A	109.00
C31—C30—H30	119.00	C71—C72—H72B	109.00
C32—C31—H31	121.00	C71—C72—H72C	109.00
C30—C31—H31	121.00	H72A—C72—H72B	110.00
C33—C32—H32	119.00	H72A—C72—H72C	110.00
C31—C32—H32	119.00	H72B—C72—H72C	109.00
			
C9—N1—C1—C2	2.3 (8)	C22—C23—C24—C25	−1.8 (9)
C9—N1—C1—C11	−173.9 (5)	C23—C24—C25—C26	0.9 (10)
C1—N1—C9—C4	−2.0 (8)	C24—C25—C26—C27	0.9 (9)
C1—N1—C9—C8	177.4 (5)	C25—C26—C27—N3	−179.1 (5)
C17—N2—C16—C11	136.2 (6)	C25—C26—C27—C22	−1.9 (9)
C17—N2—C16—C15	−42.2 (8)	C30—C29—C34—N4	178.0 (5)
C16—N2—C17—O1	−2.1 (10)	C34—C29—C30—C31	3.1 (10)
C16—N2—C17—C18	−179.7 (6)	C19—C29—C34—N4	−6.1 (9)
C27—N3—C19—C20	−0.4 (8)	C30—C29—C34—C33	−2.7 (8)
C27—N3—C19—C29	−175.4 (5)	C19—C29—C34—C33	173.2 (5)
C19—N3—C27—C22	0.2 (8)	C19—C29—C30—C31	−172.9 (6)
C19—N3—C27—C26	177.4 (5)	C29—C30—C31—C32	−1.9 (11)
C35—N4—C34—C33	−42.6 (8)	C30—C31—C32—C33	0.2 (11)
C34—N4—C35—O2	−1.0 (10)	C31—C32—C33—C34	0.2 (10)
C35—N4—C34—C29	136.7 (6)	C32—C33—C34—N4	−179.6 (6)
C34—N4—C35—C36	−179.7 (6)	C32—C33—C34—C29	1.1 (9)
C37—N5—C45—C44	−176.9 (5)	C47—C37—C38—C39	−176.5 (5)
C37—N5—C45—C40	1.9 (8)	N5—C37—C47—C48	151.7 (6)
C45—N5—C37—C47	174.4 (5)	N5—C37—C38—C39	−0.3 (8)
C45—N5—C37—C38	−1.9 (7)	C38—C37—C47—C52	145.0 (6)
C53—N6—C52—C47	−135.1 (6)	N5—C37—C47—C52	−31.3 (8)
C52—N6—C53—O3	−3.6 (10)	C38—C37—C47—C48	−32.0 (8)
C53—N6—C52—C51	43.5 (9)	C37—C38—C39—C40	2.4 (9)
C52—N6—C53—C54	174.8 (6)	C37—C38—C39—C46	178.2 (5)
C55—N7—C63—C58	1.6 (8)	C46—C39—C40—C45	−178.1 (6)
C55—N7—C63—C62	−177.6 (5)	C38—C39—C40—C41	177.8 (6)
C63—N7—C55—C65	174.9 (5)	C38—C39—C40—C45	−2.3 (8)
C63—N7—C55—C56	−1.9 (8)	C46—C39—C40—C41	2.1 (9)
C70—N8—C71—C72	175.2 (6)	C45—C40—C41—C42	0.4 (9)
C71—N8—C70—C65	−135.5 (6)	C39—C40—C45—N5	0.2 (8)
C70—N8—C71—O4	−1.8 (10)	C39—C40—C45—C44	179.0 (5)
C71—N8—C70—C69	42.6 (9)	C41—C40—C45—N5	−179.9 (5)
C11—C1—C2—C3	174.8 (5)	C41—C40—C45—C44	−1.1 (8)
N1—C1—C11—C12	−152.0 (6)	C39—C40—C41—C42	−179.8 (6)
C2—C1—C11—C12	31.8 (8)	C40—C41—C42—C43	0.7 (10)
N1—C1—C11—C16	31.9 (8)	C41—C42—C43—C44	−0.9 (10)
C2—C1—C11—C16	−144.3 (6)	C42—C43—C44—C45	0.1 (9)
N1—C1—C2—C3	−1.4 (9)	C43—C44—C45—N5	179.8 (5)
C1—C2—C3—C4	0.0 (8)	C43—C44—C45—C40	0.9 (9)
C1—C2—C3—C10	−178.9 (6)	C37—C47—C48—C49	172.9 (6)
C10—C3—C4—C9	179.2 (5)	C52—C47—C48—C49	−4.2 (10)
C2—C3—C4—C9	0.3 (8)	C37—C47—C52—N6	5.1 (8)
C10—C3—C4—C5	1.6 (9)	C48—C47—C52—N6	−177.8 (5)
C2—C3—C4—C5	−177.3 (5)	C48—C47—C52—C51	3.6 (8)
C3—C4—C9—C8	−178.7 (5)	C37—C47—C52—C51	−173.5 (5)
C5—C4—C9—N1	178.4 (5)	C47—C48—C49—C50	2.3 (11)
C9—C4—C5—C6	0.7 (8)	C48—C49—C50—C51	0.4 (10)
C5—C4—C9—C8	−1.0 (8)	C49—C50—C51—C52	−0.9 (10)
C3—C4—C9—N1	0.7 (8)	C50—C51—C52—N6	−179.8 (6)
C3—C4—C5—C6	178.3 (6)	C50—C51—C52—C47	−1.2 (9)
C4—C5—C6—C7	0.4 (9)	N7—C55—C56—C57	1.0 (9)
C5—C6—C7—C8	−1.1 (10)	C65—C55—C56—C57	−175.7 (5)
C6—C7—C8—C9	0.8 (10)	N7—C55—C65—C66	151.4 (5)
C7—C8—C9—C4	0.2 (9)	N7—C55—C65—C70	−32.0 (7)
C7—C8—C9—N1	−179.2 (6)	C56—C55—C65—C66	−31.7 (7)
C12—C11—C16—N2	178.4 (5)	C56—C55—C65—C70	144.9 (5)
C16—C11—C12—C13	2.9 (9)	C55—C56—C57—C58	0.3 (9)
C1—C11—C16—C15	173.0 (5)	C55—C56—C57—C64	178.5 (6)
C1—C11—C16—N2	−5.4 (8)	C56—C57—C58—C59	178.5 (6)
C12—C11—C16—C15	−3.3 (8)	C56—C57—C58—C63	−0.7 (8)
C1—C11—C12—C13	−173.5 (6)	C64—C57—C58—C59	0.3 (9)
C11—C12—C13—C14	−1.2 (10)	C64—C57—C58—C63	−178.9 (5)
C12—C13—C14—C15	−0.2 (10)	C57—C58—C59—C60	−179.1 (6)
C13—C14—C15—C16	−0.2 (10)	C63—C58—C59—C60	0.0 (9)
C14—C15—C16—C11	2.0 (9)	C57—C58—C63—N7	−0.3 (8)
C14—C15—C16—N2	−179.6 (6)	C57—C58—C63—C62	178.9 (5)
N3—C19—C20—C21	1.2 (9)	C59—C58—C63—N7	−179.5 (5)
C29—C19—C20—C21	176.2 (5)	C59—C58—C63—C62	−0.3 (8)
C20—C19—C29—C34	−142.6 (6)	C58—C59—C60—C61	0.2 (10)
C20—C19—C29—C30	33.1 (8)	C59—C60—C61—C62	0.0 (9)
N3—C19—C29—C30	−151.7 (6)	C60—C61—C62—C63	−0.3 (9)
N3—C19—C29—C34	32.5 (8)	C61—C62—C63—N7	179.7 (5)
C19—C20—C21—C22	−1.7 (9)	C61—C62—C63—C58	0.5 (9)
C19—C20—C21—C28	−179.2 (6)	C55—C65—C66—C67	173.5 (6)
C28—C21—C22—C27	179.0 (6)	C70—C65—C66—C67	−3.3 (9)
C28—C21—C22—C23	0.2 (9)	C55—C65—C70—N8	4.7 (8)
C20—C21—C22—C23	−177.3 (6)	C55—C65—C70—C69	−173.5 (5)
C20—C21—C22—C27	1.5 (8)	C66—C65—C70—N8	−178.7 (5)
C21—C22—C23—C24	179.6 (6)	C66—C65—C70—C69	3.2 (8)
C21—C22—C27—C26	−177.8 (5)	C65—C66—C67—C68	1.4 (10)
C27—C22—C23—C24	0.9 (9)	C66—C67—C68—C69	0.7 (10)
C21—C22—C27—N3	−0.7 (8)	C67—C68—C69—C70	−0.8 (10)
C23—C22—C27—C26	1.0 (9)	C68—C69—C70—N8	−179.4 (6)
C23—C22—C27—N3	178.1 (5)	C68—C69—C70—C65	−1.2 (9)

Symmetry codes: (i) *x*−1, *y*−1, *z*; (ii) *x*, *y*+1, *z*; (iii) *x*−1, *y*, *z*; (iv) *x*, *y*−1, *z*; (v) *x*−1, *y*+1, *z*; (vi) *x*+1, *y*, *z*; (vii) *x*+1, *y*+1, *z*; (viii) *x*+1, *y*−1, *z*; (ix) *x*, *y*−1, *z*−1; (x) *x*, *y*, *z*+1; (xi) *x*, *y*+1, *z*+1; (xii) *x*−1, *y*, *z*−1; (xiii) *x*, *y*, *z*−1; (xiv) *x*+1, *y*, *z*+1.

### Hydrogen-bond geometry (Å, °)

**Table:**

Cg3, Cg7, Cg11, Cg15 are the centroids of the C11–C16, C29–C34, C47–C52 and C65–C70 rings, respectively.

**Table:**

*D*—H···*A*	*D*—H	H···*A*	*D*···*A*	*D*—H···*A*
N2—H2N···O4^vi^	0.86	2.42	3.181 (6)	148
N2—H2N···N1	0.86	2.25	2.741 (7)	117
N4—H4N···O3^iv^	0.86	2.39	3.160 (6)	149
N4—H4N···N3	0.86	2.25	2.735 (7)	116
N6—H6N···O2^vii^	0.86	2.51	3.262 (6)	147
N6—H6N···N5	0.86	2.21	2.703 (7)	116
N8—H8N···O1	0.86	2.49	3.239 (6)	146
N8—H8N···N7	0.86	2.20	2.705 (7)	118
C15—H15···O1	0.93	2.51	2.905 (7)	106
C33—H33···O2	0.93	2.51	2.894 (7)	105
C36—H36B···O3^iv^	0.96	2.31	3.201 (7)	154
C44—H44···O2^vii^	0.93	2.60	3.472 (9)	157
C51—H51···O3	0.93	2.53	2.927 (7)	106
C54—H54B···O2^vii^	0.96	2.28	3.213 (8)	162
C69—H69···O4	0.93	2.52	2.911 (7)	106
C10—H10A···Cg15^viii^	0.96	2.76	3.586 (7)	145
C28—H28B···Cg11	0.96	2.63	3.579 (7)	171
C46—H46C···Cg7^vi^	0.96	2.65	3.594 (7)	168
C64—H64C···Cg3^ii^	0.96	2.65	3.604 (7)	173

Symmetry codes: (vi) *x*+1, *y*, *z*; (iv) *x*, *y*−1, *z*; (vii) *x*+1, *y*+1, *z*; (viii) *x*+1, *y*−1, *z*;  (ii) *x*, *y*+1, *z*.
